# Supporting nonlinear careers to diversify science

**DOI:** 10.1371/journal.pbio.3002291

**Published:** 2023-09-14

**Authors:** Anna L. Vlasits, Monique L. Smith, Maria Maldonado, Simone Brixius-Anderko

**Affiliations:** 1 Department of Neurobiology, Northwestern University, Evanston, Illinois, United States of America; 2 Department of Neurobiology, Department of Neurosciences, University of California, San Diego, California, United States of America; 3 Department of Plant Biology, University of California, Davis, California, United States of America; 4 Department of Pharmaceutical Sciences, School of Pharmacy, University of Pittsburgh, Pennsylvania, United States of America

## Abstract

Those who follow non-linear career trajectories often face disadvantages in academia. This Perspective looks at why individuals might choose non-linear careers and how these benefit diversity in science.

## Teaser

Nonlinear careers through academia are increasingly common, but funding agencies and search committees penalize these paths. Why do scientists stray from the beaten path, how do they contribute to science, and how do we level the playing field?

The institutional structure of an academic career discourages following a nonlinear career trajectory. Most critically, time and age restrictions for early-career funding can systematically disadvantage those who have taken nonlinear career paths. We are a group of early-stage investigators working in the United States who have taken nonlinear paths through our academic careers: a high school dropout who restarted her education after having children; an immigrant scientist who left academia to work as a consultant and financial analyst for an extended period and then returned; a mother who started her family during her PhD and left academia when the career became incompatible with parenthood, only to return later; and a first-generation college student from a low socioeconomic background who worked full time throughout her education. Here, we argue that those with more diverse career histories benefit the scientific community. We propose better practices for targeting funding to early-career scientists without creating obstacles for those who have taken nonlinear career paths.

Obtaining funding to support a lab is critical for establishing an early-stage investigator’s career. In recognition of the fact that early-stage investigators may not be able to compete effectively against established investigators, many funders specifically target early-stage investigators, aiming to kickstart their careers. To equalize the playing field, these funders have eligibility criteria. For instance, the largest funder of STEM research in the world, the US National Institutes of Health (NIH) [1], restricts early-stage investigator status to researchers who are within 10 years of their terminal research degree [2]. Another common requirement is an age limit. While the majority of public and private programs in the US allow for exceptions, these are usually inflexible and limited to medical, caregiving, military, or natural-disaster situations. Notably, the NIH explicitly does not consider “career choices,” such as time away from research in non-research-oriented industries, as the basis for an extension request [3]. These eligibility criteria have a substantial effect on the wider funding landscape, as many private foundations in the US base their policies on NIH standards.

**Fig 1:**
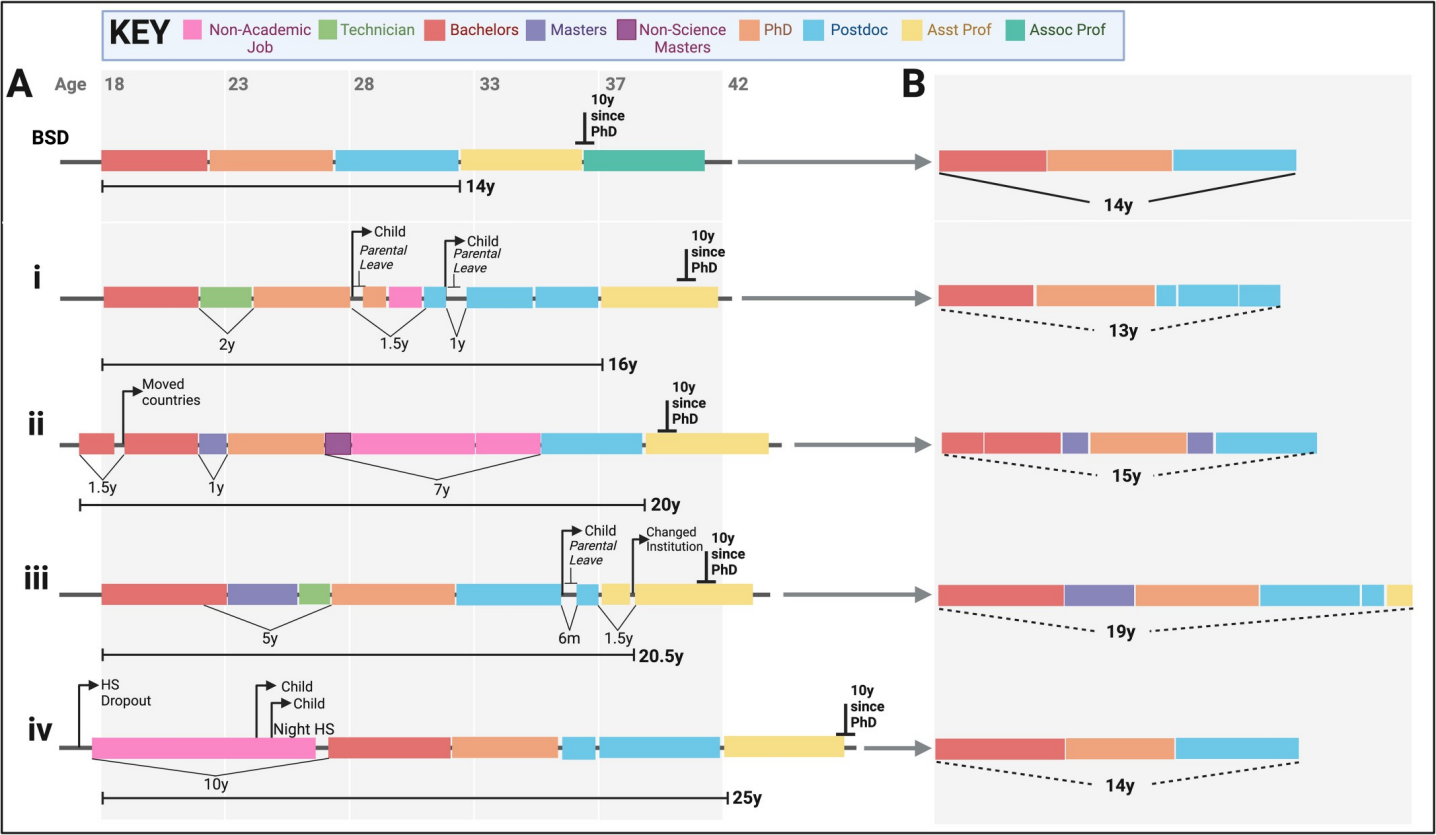
Academic trajectory comparisons to a model linear career. (A) The trajectory for fictional Big Shining Doctor (BSD) is compared to each author’s career trajectory (i–iv), including nonlinear academic and non-academic time periods. (B) Each individual career progression is shown with the nonlinear components “spliced out.” Note how timelines for i–iv are almost equivalent to BSD in panel B. Age is marked at the top and careers are color-coded by stage (see key). The 10-year mark from PhD conferral is also marked for each career. Boxes are drawn to scale with respect to time. HS, high school.

Limitations relative to absolute age, time from doctoral degree, and/or duration of postdoctoral training arbitrarily select against early-stage investigators who have taken nonlinear or nontraditional career paths. To illustrate this, we have depicted our own career paths against the “model” linear career path (Fig 1). Taking a nonlinear path can strongly diminish or even erase a junior faculty member’s eligibility for many funding sources, including the critical early-stage investigator status at the NIH. These policies are likely to disproportionately affect women, caregivers, and those from underrepresented and low socioeconomic backgrounds. Some of these criteria are ageist and many are counterproductive to funders’ stated goals of diversity, equity, and inclusion.

The reasons that a scientist may take a less straightforward path through their career are generally tied to socioeconomic inequality, elitism, and exclusion of minority populations, caregivers, and women [4]. Examining our own paths has shown how the financial burdens of education can lead to slow progress through the education period. Those coming from disadvantaged backgrounds may start their education later and take longer, accumulate student debt during undergraduate and Master’s studies, work multiple jobs, face periods of job scarcity, work outside of academia in between stages of the academic career, and experience more stressors and challenges while navigating the “hidden curriculum” of academia [5]. After the education period, graduate students and post-docs are particularly affected by low compensation, which weighs especially heavily on individuals from low socioeconomic backgrounds.

Economic factors strongly intersect with parenthood, which may also lead to a slower academic career. The low compensation at early stages of academia poses an obstacle for many caregivers, and parenthood can result in a substantial slowing of productivity given that many universities do not offer infrastructure for daycare and other child support [6]. This leaves parents with less time for their experiments, particularly if they cannot afford external support. Other reasons a scientist might leave academia for a time include caregiving (a health condition or sick family member may shelve working for a period of time), the personal or financial choice to try another career, location (they may want to live closer to family or a significant other or avoid relocating many times) [7], the desire to use their scientific education in another sector, or needing a break from academic life. Whether financial, familial, health-related, or personal, all are valid reasons to pursue a nonlinear academic path and should not create obstacles to obtaining funding at the early-faculty stage.

We believe that people who have had nonlinear career trajectories bring unique and beneficial qualities to academia. Despite their background or the life events that might have made their course less straightforward, these scientists persisted. Overcoming challenges and failure is an integral part of the academic path. Resilience requires motivational intensity and persistence, traits that are inarguably important for a successful academic career. We need to recognize that scientists with nonlinear career histories have developed a level of motivational intensity that is predictive of success in academia. The scientific community should embrace such resilience. In addition, job skills from working in other sectors are valuable and round out a faculty member’s abilities to run a lab [8]. We plan to seek out such individuals as we hire for our own labs, rewarding perseverance and welcoming diverse perspectives, which we believe will ultimately benefit academia as a whole.

To improve opportunities for career advancement and funding for people who have taken nonlinear paths, we suggest several practices that could be implemented. First, funding agencies should be intentional about their goals when setting age requirements for opportunities. In general, for faculty-level funding, the clock should start from the time that the first tenure-track-equivalent faculty position began and not from the end of the PhD or start of the post-doctoral period. Funding should not include an age cap. Agencies should recognize that many case-by-case discussions will be needed and assign resources accordingly, rather than imposing inflexible criteria. One size does not fit all.

Second, funding agencies and hiring committees in the US should standardize the ability to justify periods of low productivity or career breaks. Policies of various European funding agencies that extend eligibility based on a calculated time period worked, accounting explicitly for part-time work, family/caregiving leave, and periods employed outside of the field, may provide inspiration [9].

Third, hiring and funding groups should consider alternative strategies for gathering key career productivity information into CVs and bio-sketches (i.e., [10]) to make this material less biased (Fig 1). Scientists should be judged on their productivity during the periods when they have been working as scientists and the knowledge and competencies they bring from wider experience of other roles/sectors. Scientists should not be judged negatively for taking on other roles or making their way more slowly through their education or training.

In summary, those who have taken nonlinear paths through their academic careers are currently disadvantaged by limits to funding eligibility and negative bias for their choices. Because nonlinear paths are more likely to be taken by women, caregivers, and those from underrepresented or low socioeconomic backgrounds, many funding eligibility requirements are in conflict with the goals of the scientific community to improve representation of people from a wide variety of backgrounds in the sciences. We believe that people who have taken nonlinear paths enrich the scientific community, bringing creativity and resourcefulness as well as job skills and life experiences that benefit the lab. Academic scientists who have taken nonlinear paths through their careers deserve equitable chances at funding opportunities and career advancement.
